# A novel mechanism of phenotypic heterogeneity in Creutzfeldt-Jakob disease

**DOI:** 10.1186/s40478-020-00966-x

**Published:** 2020-06-19

**Authors:** Satish K. Nemani, Xiangzhu Xiao, Ignazio Cali, Laura Cracco, Gianfranco Puoti, Massimiliano Nigro, Jody Lavrich, Anuradha Bharara Singh, Brian S. Appleby, Valerie L. Sim, Silvio Notari, Witold K. Surewicz, Pierluigi Gambetti

**Affiliations:** 1 Department of Pathology, Case Western Reserve University, School of Medicine, Cleveland, OH 44106 USA; 2 Department of Physiology and Biophysics, Case Western Reserve University, School of Medicine, Cleveland, OH 44106 USA; 3 National Prion Disease Pathology Surveillance Center, Case Western Reserve University, School of Medicine, Cleveland, OH 44106 USA; 4 Department of Advanced Medical and Surgical Sciences, University of Campania “L. Vanvitelli”, 81100 Caserta, Italy; 5 Department of Mental Health and Emergency Psychiatry, P. O. “Maresca”, Asl Napoli 3 Sud, 80059 Torre del Greco, Italy; 6 Vascular Medicine Institute, University of Pittsburgh, Pittsburgh, PA 15261 USA; 7 Department of Neurology, Case Western Reserve University, School of Medicine, Cleveland, OH 44106 USA; 8 Department of Psychiatry, Case Western Reserve University, Cleveland, OH 44106 USA; 9 Centre for Prions and Protein Folding Diseases, University of Alberta, Edmonton, T6G2M8 Canada

**Keywords:** Prion protein allotype, 129 M and 129 V proportion, 129 M/V polymorphism, Epitope mapping, Mass spectrometry

## Abstract

One of remarkable features of sporadic Creutzfeldt-Jakob disease (sCJD) is the great phenotypic variability. Understanding the molecular basis of this variability has important implications for the development of therapeutic approaches. It is well established that, in many cases, phenotypic heterogeneity of sCJD is under control of two determinants: the genotype at the methionine (M)/valine (V) polymorphic codon 129 of the human prion protein gene and the type, 1 or 2, of the pathogenic and disease-related form of the prion protein, PrP^D^. However, this scenario fails to explain the existence of distinct heterozygous sCJDMV2 subtypes, where heterogeneity occurs without any variation of the 129 allotype and PrP^D^ type. One of these subtypes, denoted sCJDMV2C, associated with PrP^D^ type 2, is characterized by widespread spongiform degeneration of the cerebral cortex (C). The second variant, denoted sCJDMV2K, features prominent deposition of PrP^D^ amyloid forming kuru type (K) plaques. Here we used a mass spectrometry based approach to test the hypothesis that phenotypic variability within the sCJDMV2 subtype is at least partly determined by the abundance of 129 M and 129 V polymorphic forms of proteinase K-resistant PrP^D^ (resPrP^D^). Consistent with this hypothesis, our data demonstrated a strong correlation of the MV2C and MV2K phenotypes with the relative populations of protease-resistant forms of the pathogenic prion proteins, resPrP^D^-129 M and resPrP^D^-129 V, where resPrP^D^-129 M dominated in the sCJDMV2C variant and resPrP^D^-129 V in the sCJDMV2K variant. This finding suggests an important, previously unrecognized mechanism for phenotypic determination in human prion diseases.

## Introduction

The variability of the clinical presentation in Creutzfeldt-Jakob disease (CJD) has been known for many years and has led to the identification of several clinical variants [5]. In subsequent years, we showed that the phenotypic heterogeneity of the sporadic form of CJD (sCJD) is largely driven by the pairing of two sets of major determinants of disease phenotype: the genotype – MM, MV, VV – at the methionine (M)/ valine (V) polymorphic codon 129 of the human prion protein (PrP) gene and the type, 1 or 2, of the pathogenic, disease-related form of the prion protein (PrP^D^) [12, 24, 25, 27, 29]. PrP^D^ type 1 and 2, which were initially distinguished by the electrophoretic mobilities as species with 21 kDa (type 1) and 19 kDa (type 2) proteinase-resistant cores, resPrP^D^, of the unglycosylated forms, were shown to represent two fundamental prion strains associated with sCJD [3, 29, 31]. This concept led to a new classification of sCJD that theoretically included six phenotypes or subtypes: MM, MV and VV, each of which could be associated with PrP^D^ type 1 or 2. Indeed, as expected, sCJD patients with the 129 MM genotype coupled with PrP^D^ type 1, commonly identified as sCJDMM1 (hereafter MM1), as well as sCJDVV2, VV1 and MV2 were associated with distinct disease phenotypes (Table 1). Some of these phenotypes mimicked the clinical variants originally proposed, while some were novel. However, detailed analyses revealed two apparent inconsistencies. First, MM1 and MV1 were shown to share histopathological phenotype and PrP^D^ electrophoretic profile, prompting their grouping into one sCJD subtype, namely MM (MV)1 [12, 27]. Second, MM2 was associated with two quite distinct phenotypes: one with sCJD-like features including severe spongiform degeneration (SD) of the cerebral cortex, named sCJDMM2 (or MM2C for cortical), and the other, very rare, characterized by insomnia and severe thalamic atrophy that is commonly referred to as sporadic fatal insomnia (sFI or MM2T for thalamic) [8]. Specific variations of PrP^D^ as well as distinct transmission characteristics indicate that MM2 and sFI are associated with distinct prion strains [3, 9, 19, 26].

**Table 1:** Synopsis of molecular classification and phenotypes of sCJD subtypes and variants

sCJD subtype^**a**^; Prevalence (%)	Histopathological phenotype	WB resPrP^**D**^ Profile
MV2K; ~ 8	SD pseudo-laminar in CC (Fig. 1a-d); kuru pl. in Cbl. IHC: fine punctate in CC; Cbl (Fig. 1a-d)	Type 2 (19 kDa) + Type ~ 1 (20 kDa)
MV2C; ~ 2	SD: large confluent vacuoles mostly in CC. IHC: co-distributing with SD (Fig. 1e-h).	Type 2 (19 kDa)
MV1; 5	SD with fine vacuoles predominantly in CC; IHC: fine punctate pattern in SD regions; in Cbl “brushstroke” pattern (Fig. 1s-u)	Type 1 (21 kDa)
VV2; 15	SD like MV2K in CC; no kuru plaques but plaque-like aggregates in Cbl (Not shown)	Type 2 (19 kDa)
MM2; 4	As MV2C (Fig. 1i-l)	Similar but not identical to MV2C
MM1; 64	As MV1 (Fig. 1v-x)	As MV1

^a^Subtype terminology: M (methionine) and V (valine) refer to the genotype at PrP codon 129; 1 and 2 refer to PrP^D^ types 1 and 2, which along with the 129 genotype are major determinants of the disease phenotype; the C in MV2C refers to the severe involvement of the cerebral cortex in this condition as in MM2; K in MV2K refers to the presence of kuru plaques. *Abbreviations*: *CC* cerebral cortex, *SD* spongiform degeneration, *IHC* PrP immunohistochemistry. Figure 1 panels illustrate the features of SD and IHC patterns. The VV1 subtype (2% prevalence) has been omitted

Recently, the issue of phenotypic heterogeneity within the MV2 subtype has also emerged [23, 28] (Table 1). The MV2 subtype was shown to comprise two basic histopathological phenotypes (or histotypes) as well as two electrophoretic profiles of resPrP^D^: a variant that was indistinguishable from the MM2 subtype (named sCJDMV2C) and a second phenotype that, although generally mimicking sCJDVV2, differed from the latter with regard to the prominent presence of kuru plaques (MV2K) as well as the presence of a small additional resPrP^D^ component in the otherwise type 2 resPrP^D^ profile. The coexistence in the same case of the two histotypes in various proportions (MV2K-C) was also observed.

In 2016, Moore et al. carried out a mass spectrometric study to determine the relative amount of resPrP^D^-129 M and -129 V in PrP^D^ enriched preparations extracted from MV1, MV2C and MV2K cases as well as cases with mixed histotypes, such as MV2K-C and MV1-2C [20]. Based on the finding that the ratios of resPrP^D^-129 M and -129 V allotypes were highly variable, the authors concluded that, in MV2 and MV1 cases, normal or cellular PrP (PrP^C^)-129 M and -129 V had “similar tendency to misfold” into PrP^D^ regardless of the MV histotype, and that “factors other than the PrP^Sc^ allotype abundance must influence the clinical progression and the phenotype of heterozygous cases of CJD”.

Here, we have re-examined the issue of resPrP^D^-129 M and -129 V relative abundance in the sCJDMV case subset using mass spectrometry. Given the variability, sometimes subtle, of the phenotype in these cases, we extensively analyzed the histotype and PrP^D^ characteristics to select MV2C and MV2K cases associated with only one phenotype (called “pure”), along with MV2K-C mixed cases, where the two histotypes coexisted in well-defined proportions. In all MV2 cases examined, we consistently observed a positive correlation between the relative dosage of resPrP^D^-129 M and -129 V allotypes and the MV2C and MV2K histotypes. However, surprisingly, in the MV1 variant, resPrP^D^-129 M and -129 V were equally represented.

## Materials and methods

### Brain tissues

All frozen and fixed brain tissues were obtained from the National Prion Disease Pathology Surveillance Center (NPDPSC) at Case Western Reserve University (CWRU) in Cleveland, OH. A cohort of 23 subjects was selected that, according to common sCJD subtype classifications, comprised 21 cases harboring exclusively one histotype, and 2 cases with mixtures of two co-existing histotypes [1, 27]. The 21 sCJD “pure” cases cohort included: 4 MM1; 3 MV1; 4 MM2; 4 MV2C; 3 MV2K; 3 VV2. Three cases of pure MV2C, MV2K, and MV1 each and two cases in which the histotype was mixed were used for mass spectrometry analyses. In one of the mixed cases, the MV2K and MV2C histotypes co-existed in equal ratio (50:50); in the other one, the cerebral cortex showed pure MV2C histotype while the mixed MV2K + C histotype with a 90:10 ratio was observed in the cerebellar cortex.

### Reagents and antibodies

Tris-buffered saline (TBS), Tris/glycine buffer, Tris/glycine/SDS buffer, Tween 20, β–mercaptoethanol, Dithiothreitol (DTT), Laemmli sample buffer, Non-fat dry milk, 15% Criterion Tris-HCl polyacrylamide gels were procured from Bio-Rad Laboratories (Hercules, CA, USA). Benzonase, Polyvinylidene difluoride (PVDF) (Immobilion-P and Immobilon-FL) were purchased from Millipore Sigma (Burlington, MA, USA). 1 M Tris-HCl, N-Lauroylsarcosine sodium salt solution, 3- Indole acetic acid, 8 M Guanidine-hydrochloride, (GdnHCl), Calcium chloride, Phenylmethylsulfonyl fluoride (PMSF), Dulbecco’s Phosphate Buffered Saline (D-PBS), Proteinase K, Iodoacetamide (IAA) and Ammonium bicarbonate were obtained from Sigma-Aldrich. NuPAGE 4–12% Bis-Tris gradient gels, NuPAGE MES SDS running buffer (20X), Acetonitrile, Silver Staining kit, Ethanol, Formic Acid, Methanol, Pierce ECL 2 Western Blotting Substrate, Odyssey Blocking Buffer and Trypsin from Thermo Fisher Scientific Inc. (Waltham, MA, USA). The following antibodies were used: 3F4 (to human PrP residues 106–110) [15], 12B2 (to human PrP residues 89–93) [18, 32], Tohoku-2, polyclonal antibody (to residues 97–103) [17] and SAF32 (to human prion octapeptide repeat region 59–89) [11], Alkaline phosphatase-conjugated goat anti mouse or anti rabbit (Promega, Madison, WI, US), Horseradish peroxidase (HRP)- conjugated sheep anti-mouse or donkey anti-rabbit IgG Ab sheep anti-mouse IgG (GE Healthcare Life Sciences, Chicago, IL, USA) and Infrared Dye (IRDye) 800CW goat anti-mouse IgG (LI-COR Biosciences, Lincoln, NE, USA).

### Molecular genetics

DNA was isolated from frozen brain tissues and genotyping of *PRNP* coding region was performed as previously described [25].

### Histopathology

Histopathology and immunohistochemistry were performed essentially as described previously [5, 6].

### PK-resistant PrP^D^ analysis

Brain homogenates (BH) (10% *w*/*v*) were prepared as previously described [22]. Brain homogenate was centrifuged at 1000 x g for 10 min and supernatant (S1) was collected. S1 were treated with 70 U/ml proteinase K (PK) and incubated at 37 °C for 1 h. Protease digestion was stopped by adding 4 mM PMSF. Samples were mixed with equal volumes of 2X sample buffer (6% SDS, 8% 2-mercaptoethanol, 20% glycerol, 4 mM EDTA, 125 mM Tris HCl pH 6.8), boiled for 10 min and incubated with 8-fold pre-chilled methanol overnight at − 20 °C. The following day, the samples were centrifuged at 18,200 x g for 30 min, pellets were resuspended in 1X sample buffer by sonication and subjected to immunoblotting.

Protein samples (brain tissue equivalent 0.1–3 mg of wet tissue) were separated in 4–12% NuPAGE Bis-Tris gradient gels and blotted into Immobilon-FL membranes for 40 min at 30 V, blocked with 5% nonfat dry milk in TBS-T (1× TBS with 0.1% Tween 20) and probed with the indicated antibodies. Immunoblots were visualized by ImageQuant LAS 4000 or by Odyssey infrared imaging system (LICOR Biosciences) as described by the manufacturer.

### Conformational stability immunoassay (CSI)

CSI was performed according to previous protocols [4, 30, 35] with slight modifications. Aliquots of BH (10% *w*/*v*) prepared in LB100 (pH 8.0) were centrifuged at 1000 × g for 10 min at 4 °C and the S1 fraction were collected. Fifty μl aliquot of S1 was diluted with an equal volume of GdnHCl to obtain final GdnHCl concentrations ranging from 0 to 3.5 M and samples were incubated for 1.5 h at 22 °C. GdnHCl was subsequently removed by incubating each sample with an excess of 8-fold pre-chilled methanol overnight at − 20 °C, followed by centrifugation at 18,200 × g for 30 min. Pellets were resuspended in 50 μl LB100 (pH 8.0) by sonication. Each aliquot was digested with 10 U/ml PK for 1 h at 37 °C and reaction was terminated by addition of 4 mM PMSF. Samples were denatured and loaded onto 15% precast Tris-HCl gels. PrP amounts at different GdnHCl concentrations were measured by Odyssey application software V3.0. The conformational stability curves (i.e., fraction of remaining PrP^D^ as a function of GdnHCl concentration) for unglycosylated PrP^D^ were fitted to a sigmoidal equation using GraphPad Prism. The mean [GdnHCl]_1/2_ values ± standard error of the mean (SEM) were calculated and compared between histotype case groups.

### Proteinase K titration assay

The proteinase K (PK) titration assay was performed as described previously [7]. Briefly, 30 μl of S1 was treated with different concentrations of PK (0.62, 1.25, 2.5, 5, 10, 20, 40, 80, 160 U/ml) for 1 h at 37 °C. Additionally, S1 was incubated with 320 U/ml of PK for 12 h at 37 °C. The PK digestion was terminated with 4 mM PMSF; equal aliquots of 2X sample buffer were added and samples were boiled for 10 min and subjected to immunoblotting with 3F4. The PK points from 5 to 320 U/ml of the unglycosylated resPrP^D^ fragment from sCJD variants were best fitted using one phase decay and PK_½_ index values ± SEM were determined and compared between subtypes.

### Purification of resPrP^D^

Purification of resPrP^D^ was performed as previously reported [10] following extraction from the cerebral cortex (CC) of sCJDMV1, MM2, MV2C, MV2K, VV2 and and MV2K-C cases. In a mixed case containing the sCJDMV2C histotype in the cerebral cortex and the sCJDMV2K in the cerebellum, resPrP^D^ was purified also from the cerebellar cortex.

### Silver staining

Purity of resPrP^D^ corresponding to different sCJD subtypes was analyzed by gel electrophoresis with silver staining detection according to manufacturer instructions (Thermo Fisher Scientific Inc., Waltham, MA, USA).

### In-solution digestion of purified resPrP^D^

In-solution trypsin digestion was performed according to the previously method [14] with some modifications. Briefly, purified resPrP^D^ samples were dissociated by incubation with 8 M GdnHCl for 10 min; this was followed by reduction with DTT and alkylation with IAA step. After centrifugation, the supernatant was collected and incubated overnight at − 20 °C with nine volumes of methanol to precipitate the protein. The precipitates were pelleted by centrifugation at 21,130 x g for 30 min at 4 °C and then resuspended in 100 mM ammonium bicarbonate. Trypsin (MS grade, Thermo Scientific) was dissolved according to the manufacturer’s instructions and added to the protein solution at the enzyme to resPrP^D^ weight ratio of 1:10. The mixture was incubated overnight at 37 °C with shaking, and the reaction was terminated by adding formic acid to a final concentration of 5% (vol/vol). Samples were concentrated to a volume of ~ 20 μl and stored at − 80 °C until analysis by Nano LC-MS.

### Nano LC-MS/MS

Nanospray LC-MS-MS analysis was performed using an LTQ Orbitrap XL mass spectrometer equipped with nanoelectrospray source (Thermo Scientific, San Jose, CA). Trypsin-digested samples were loaded onto a C-18 trap column (to remove salts) and separated on a C-18 column (Acclaim PepMap, Thermo Scientific, CA) connected to an emitter. Separation was performed using a Dionex UltiMate 3000 system (Thermo Scientific, San Jose, CA) and a gradient of acetonitrile in water containing 0.1% formic acid. The flow rate was 300 nl/min. The mass spectrometer was externally calibrated using a Pierce LTQ ESI positive ion calibration solution (Thermo Scientific, catalog number 88322). MS-MS data in full scan experiments were acquired in the m/z 300–1800 range at a resolution of 30,000 (FWHM at m/z 400). The following source settings were used: spray voltage = 4.2 kV; capillary temperature = 200 °C. Data-dependent MS^n^ (*n* = 2) were acquired at ITMS using collision induced dissociation (CID); the top 14 intense ions were subjected for further fragmentation. Calculation of elemental formulae was performed on the mono-isotopic peak of each ion cluster using Xcalibur software v2.2 with a mass tolerance of 3 to 5 ppm. MS/MS raw files were searched using MASCOT Deamon engine against the database containing sequence of human prion protein 129 M/V. Trypsin/P search parameters for Mascot peptide identification included one missed tryptic cleavage, fixed carbamidomethylation (+ 57 Da, Cys), and variable oxidation (+ 16 Da, Met). Mass tolerances of 2.0 and 1.0 Da were used for parent and monoisotopic fragment ions, respectively. The resulting files generated by MASCOT were used for peptide identification with the constraints that only MASCOT ion scores greater than 10 were considered. The percentage of 129 M and 129 V PrP in resPrP^D^ samples was calculated by the spectral counting method [2].

### Calibration curves for allotypic ratio determination

To obtain a calibration curve, recombinant full-length human 129 M and 129 V PrP were purified as described previously [21, 33] and the concentration of each protein was determined by absorbance at 280 nm using the extinction coefficient of 57,995 M^− 1^ cm^− 1^. Both proteins were combined at the 129 M PrP to 129 V PrP molar ratios of 0:100, 25:75, 50:50, 75:25, and 100:0. After in-solution trypsin digestion, the samples were analyzed by mass spectrometry to determine the relative ratio of the 129 M and 129 V polymorphs as described above. A similar calibration curve was also obtained using purified human resPrP^D^ from sCJDMM2 and sCJDVV2 subtypes by combining the proteins at the same the molar ratios. For this purpose, resPrP^D^ from sCJDMM2 and sCJDVV2 subtypes were quantified from Western blots by densitometry using the Odyssey application software V3.0. Recombinant PrP was used as a standard for this Western blot-based quantification.

### Statistical analysis

Statistical significance was assessed with the one-way analysis of variance (ANOVA) followed by multiple pairwise comparisons with the Tukey test supported by GraphPad Prism 8.0.

## Results

### Histopathology and immunohistochemistry

**Fig. 1:**
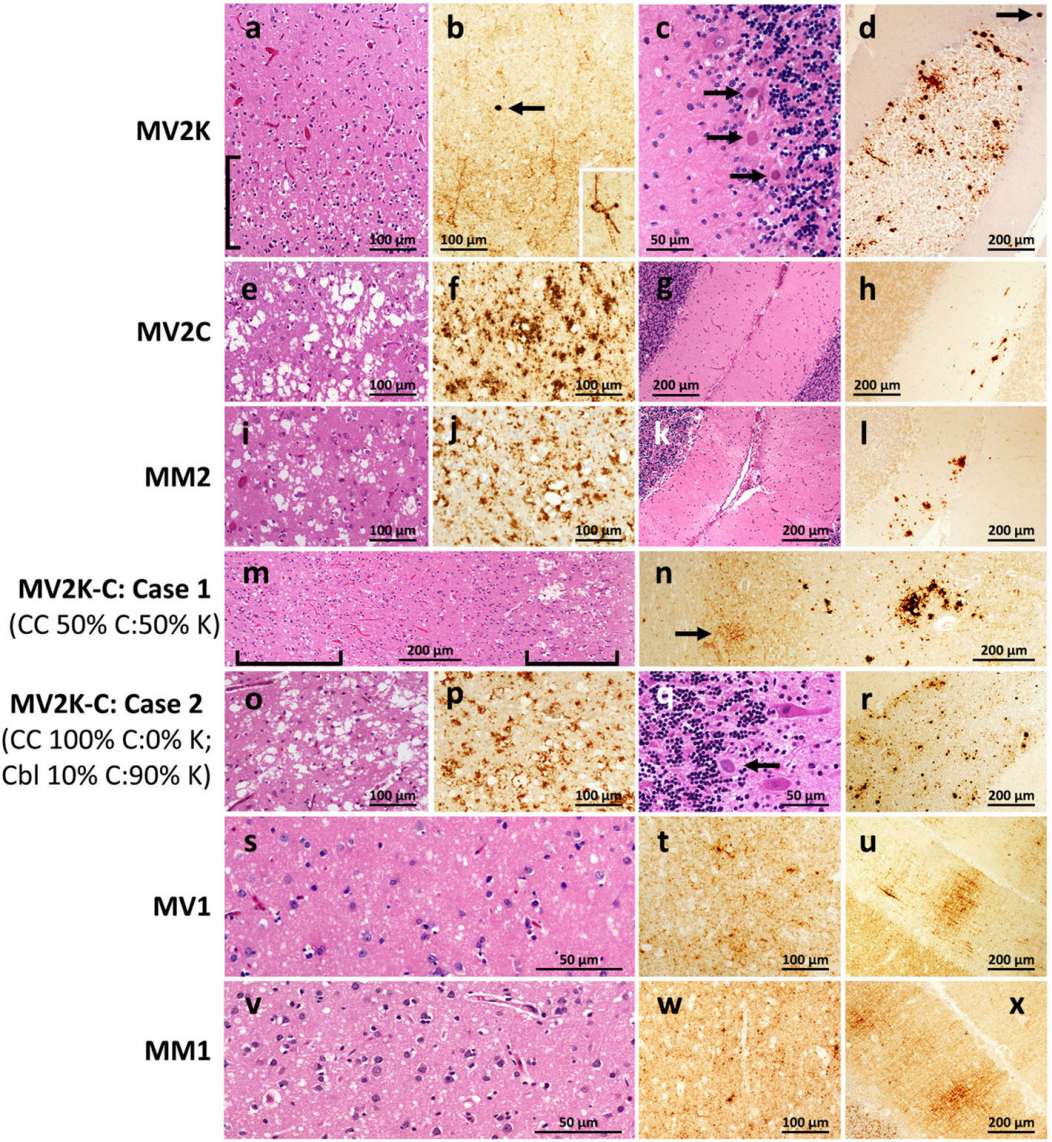
Histotypes of the sCJDMV2 variants, sCJDMV1 and controls selected for this study. Overall, MV2K subtype (**a-d**) uniquely features the presence of many kuru plaques in the cerebellum while the cerebral cortex harbors a fine vacuole laminar spongiform degeneration (SD) similar to that associated with VV2; **a** fine-vacuole, laminar SD ([) in the cerebral cortex (CC); **b** the cortical region displaying the SD is also more intensely immunostained and shows the characteristic immunostaining of the outer regions of the neuronal bodies and processes (inset) as well as occasional plaque or plaque-like deposits (arrow); **c** and **d** three kuru plaques lying at the edge of the granule cell layer of the cerebellar cortex (Cbl) (**c**) while at lower magnification many plaques are detectable over the granule cell layer, and, occasionally, in the molecular layer (arrow), by immunostaining (**d**). The MV2C histotype (**e-h**) closely resembles that of MM2 (**i-l**) and is characterized by widespread SD made of large-vacuole especially in the cerebral cortex (**e** and **i**) and is intensely immunostained (**f** and **j**) while the cerebellum is lightly or not affected (**g** and **k**) and shows focal immunostaining with large granule and plaque-like formation (**h** and **l**). Mixed sCJDMV2K-C cases (**m-r**) harbor both histotypes in variable ratios that may share the same brain regions or coexist separately; case 1 (**m** and **n**) exhibits a ~ 50:50% mixed K and C histotypes in the CC distinguishable by the co-existence of fine- vacuole with laminar distribution (left, lower bracket) and large-vacuole, grape-like SD (right, upper bracket); immunostaining (**n**) further distinguishes the two characteristic patterns: light perineuronal (arrow) and very intense staining of vacuole clusters that are the characteristics of the K and C components of sCJDMV2K-C; compare **m** with **a** and **e**, and **n** with **b** and **f**, respectively. Case 2 (**o-r**) harbored exclusively the MV2C histotype in CC (**o** and **p**) along with the MV2K histotype in ~ 90% of Cbl (**q** and **r**); arrow in **q** points to a kuru plaque while many plaques can be seen at lower magnification after immunostaining (**r**). sCJDMV1 (**s-u**) and -MM1 (**v-x**) shared the widespread and fine vacuole SD (**s** and **v**) and the same pattern of PrP^D^ aggregate formation in CC (**t** and **w**) as well as the “brush stroke-like” pattern in Cbl (**u** and **x**). Bars indicate magnifications; histological staining H.E.; immunostaining with Ab 3F4 to PrP

Detailed histopathological evaluation, including immunohistochemical and semiquantitative assessments of the individual lesions and staining patterns, led to the selection of three representative cases of pure MV2C, MV2K and MV1 histotypes (Fig. 1, panels a-n and s-x, and Table 1). We also selected two cases of MV2K-C where the MV2C and MV2K histotypes were mixed in different proportions. One of these cases was characterized by approximately equal proportion of the two histotypes throughout the brain. In the other case, the cerebral cortex harbored only the MV2C histotype, whereas the cerebellar cortex contained both histotypes, with a MV2K and MV2C ratio of ~ 9:1 (Fig. 1, panels n-r).

### Immunoblotting and epitope mapping

**Fig. 2:**
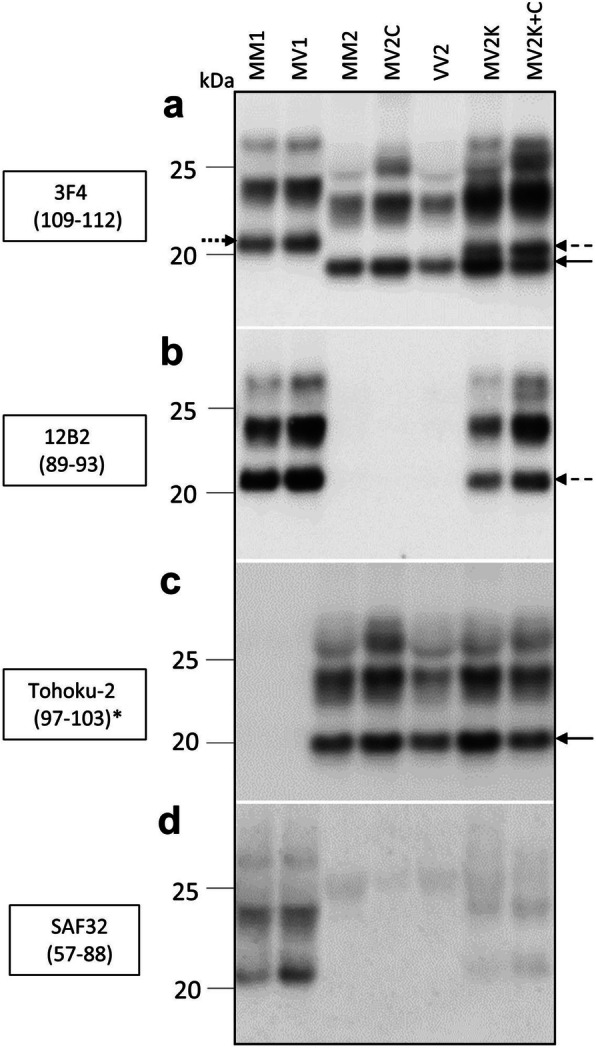
Immunoblot and N-terminus epitope mapping of resPrP^D^ from sCJDMV2 variants, sCJDMV1 and controls. **a** both resPrP^D^ types 1 and 2 (arrows) from all MV variants reacted with the type-non-specific Ab 3F4. The three resPrP^D^ major glycoform are present in all preparations. The unglycosylated components shows the ~ 21 kDa mobility of resPrP^D^ type 1 in -MM1 and -MV1 (dotted arrow), and the 19 kDa mobility of resPrP^D^ type 2 (solid arrow) in all other preparations. Both “pure” sCJDMV2K and the mixed variant MV2K-C (50:50%) exclusively display the 20 kDa band (dashed arrow) along with the 19 kDa type 2 component (solid arrow). **b** type 1-specific Ab 12B2, besides resPrP^D^ type 1 of MM1 and MV1, also demonstrated all three glycoforms of the 20 kDa component in sCJDMV2K and MV2K-C (dashed arrow) confirming the type 1-like N-terminus immunoreactivity of the 20 kDa as well as of its other two glycoform components; note that unlike with 3F4, when probing with Ab 12B2 the “20” kDa band (dashed arrow) aligns with the unglycosylated isoform of MM1 and MV1 at 21 kDa. **c** Ab Tohoku-2, type 2-specific, reacted only with all resPrP^D^ type 2 as indicated by the 19 kDa unglycosylated isoform (solid arrow). **d** SAF32 (to slightly more N-terminal epitopes than 12B2) recognizes resPrP^D^ type 1 of MM1 and MV1 but not (or very weakly) resPrP^D^ associated with MV2K and MV2K-C. All tissue samples were obtained from the frontal cortex. ^*^ Residue 97 (or another C-terminally closely adjacent N-terminus) is required for Tohoku-2 to immunoreact [17]

Consistent with the histopathological findings, the immunoblot analysis of the selected cases and appropriate controls revealed different electrophoretic profiles of resPrP^D^ associated with distinct MV histotypes (Fig. 2, Table 1). When probed with Ab 3F4 (resPrP^D^ type non-specific), resPrP^D^ from MV2K and MV2K-C cases uniquely showed an additional 20 kDa band, which represents the unglycosylated isoform and has an electrophoretic mobility that is intermediate between 21 kDa and 19 kDa of resPrP^D^ type 1 and 2, respectively. The type 1-specific Ab 12B2 strongly reacted not only with resPrP^D^ type 1 of MM1 and MV1 preparations, but also with the above mentioned 20 kDa component. As expected, 12B2 did not recognize the type 2-specific 19 kDa band. Remarkably, when probed with Ab 12B2 the 20 kDa component appeared to co-migrate with resPrP^D^ type 1 to 21 rather than 20 kDa as it does with Ab 3F4. This finding indicates that the 20 kDa component comprises at least two resPrP^D^ populations: a small subpopulation with a more N-terminal end and 21 kDa electrophoretic mobility that is exclusively recognized by the more N-terminus Ab 12B2; by contrast, the general Ab 3F4 likely reacts with both populations but the larger 20 kDa population dictates the position of the electrophoretic band. Predictably, all type 2 resPrP^D^ subtypes (MM2, MV2C and VV2) were negative when probed with Ab 12B2 (Fig. 2b). Probing with Ab Tohoku-2 (type 2-specific) confirmed first that the “20–21” kDa resPrP^D^ fragment has resPrP^D^ N-terminus consistent with type 1 since it did not react with Tohoku-2; second, it demonstrated that the 19 kDa component coexisting with the 20–21 kDa resPrP^D^ component has type 2 N-terminus characteristics (Fig. 2c). Ab SAF32 (epitope residues 57–88) reacted with MM1 and MV1, but not (or very weakly) with MV2K resPrP^D^, confirming the more C-terminal PK cleavage of the bulk of the 20 kDa fragment (Fig. 2d). Overall, the MV2C profile was very similar, though not identical, to that of MM2, showing a slightly lower mobility of the three glycoform bands, which is clearly more pronounced for the diglycosylated band, when probed with Ab 3F4 and To-2 (Fig. 2).

### Conformational stability and PK titration assays

**Fig. 3:**
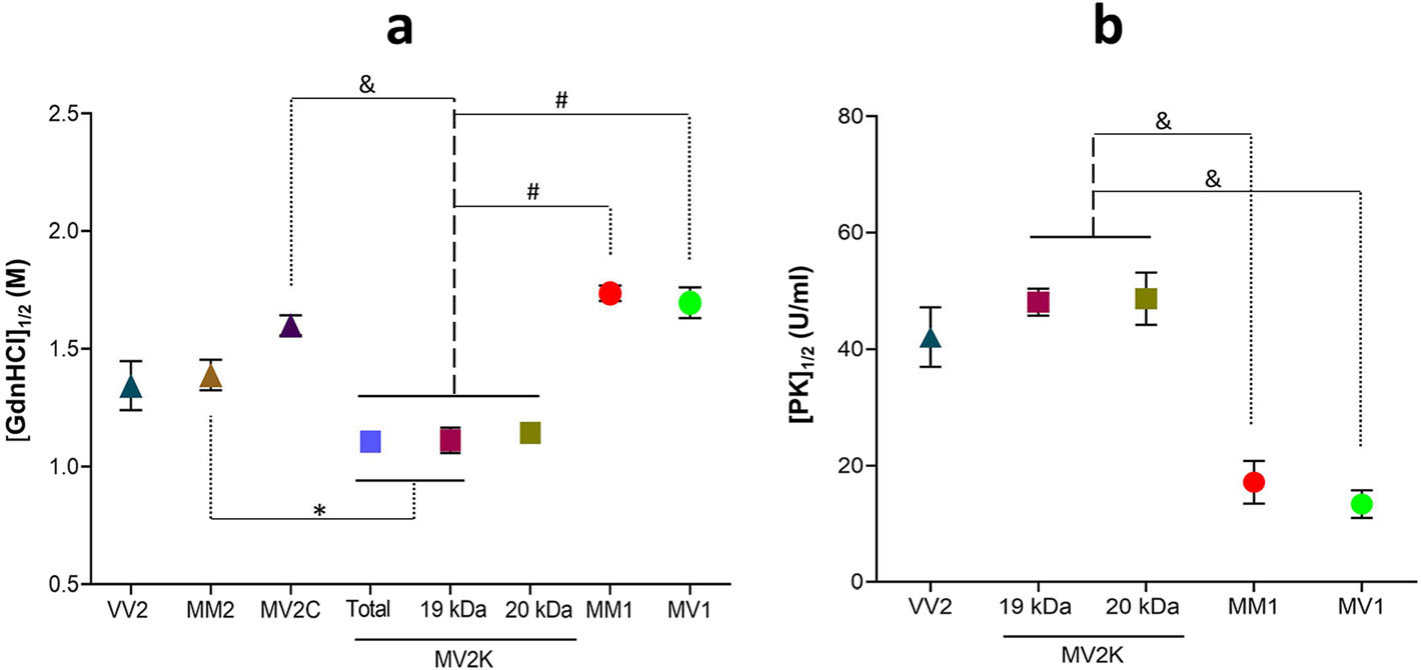
Conformational stability immunoassay (CSI) and proteinase K (PK) titration performed on “pure” sCJDMV2 variants, sCJDMV1 and sCJD subtypes used as controls. **a** coordinate graph showing the stability indexes ([GdnHCl]1/2, i.e., the concentration of GdnHCl corresponding to midpoint resPrPD denaturation) related to the individual MV2C and MV2K variants and the MV1 subtype, along with VV2, MM2 and MM1 used as sCJD subtype controls; in the MV2K variant, the stability index was determined separately for the 20 kDa and 19 kDa components, as well as combined (total). **b** coordinate graph of PK titration indexes (PK1/2, i.e., U/ml of PK corresponding to midpoint total PrP digestion) in MV2K (19 and 20 kDa components, separately), MV1 as well as VV2 and MM1 subtype controls. Both assays performed on total PrP. #: *p* < 0.0001; &: *p* < 0.0002 *p < 0.04 (See Additional File 1: Table S1 for index scores)

The conformational stability index (the concentration of GdnHCl corresponding to midpoint PrP^D^ denaturation, [GdnHCl]_1/2_) overall correlated with the histotype and resPrP^D^ electrophoretic profiles (Fig. 3a, Additional File 1: Table S1 and Additional File 3: Figure S1a). Analysis of all major sCJD subtypes uncovered a significant difference between [GdnHC]_1/2_ for MV2K and all other subtypes, especially the MV2C subtype (1.1 M vs. 1.6 M). Remarkably, the 20 kDa species, when analyzed individually, was less stable than the canonical resPrP^D^ associated with the MM1 and MV1 subtypes. As expected, no significant difference was observed between the stability of PrP^D^ associated with MM2 and MV2C subtypes, as well as between PrP^D^ associated with MM1 and MV1 subtypes, which share the histotype and electrophoretic profile (Fig. 3a, Additional File 1: Table S1 and Additional File 3: Figure S1a). Essentially similar results were obtained with the PK titration assay: again, the MV2K 20 and 19 kDa components and VV2 shared the PK_1/2_ index, which significantly differed from those of MM1, MV1 and VV1 (Fig. 3b, Additional File 1: Table S1 and Additional File 3: Figure S1b).

### Determination of relative populations of resPrP^D^-129 M and -129 V by mass spectrometry

#### Calibration of mass spectrometry-based assay

As first step in assessing the capability of our assay to accurately determine the relative population of the two resPrP^D^ allotypes in clinical samples, we performed experiments with the recombinant human PrP (rhuPrP) (Additional File 4: Figure S2a). Two polymorphic forms of the protein, rhuPrP-129 M and -129 V (concentration of which can be accurately determined by absorption spectroscopy), were combined in five different proportions and analyzed by Nano LC-MS as described in the Methods section. The relative quantities of rhuPrP-129 M and -129 V as determined by mass spectrometry correlated almost perfectly with those measured by absorption spectroscopy (*R*^*2*^ = 0.98) (Additional File 4: Figure S2a). A similar calibration was performed using resPrP^D^-129 M and -129 V purified from MM2 and VV2 cases (Additional File 4: Figure S2b) in which PrP concentration was determined by densitometry of Western blots. Peptide mapping identified multiple PrP 111–136 and PrP 111–148 fragments (Additional File 2: Table S2), which were used to determine the proportions of resPrP^D^-129 M and -129 V in five different mixtures of these samples. The relative quantities of resPrP^D^-129 M and -129 V correlated reasonably well with those determined from Western blots (Additional File 4: Figure S2b). A somewhat lower correlation (*R*^*2*^ = 0.91) in this case is likely due to the lower accuracy of densitometric analysis of Western blots compared to protein concentration determination by absorption spectroscopy. Altogether, these calibration experiments validate our mass spectrometry-based approach as an accurate tool to determine proportions of resPrP^D^-129 M and -129 V in complex clinical samples.

#### Mass spectrometric determination of resPrP^D^-129 M and -129 V proportions in MV2 subtypes

**Fig. 4:**
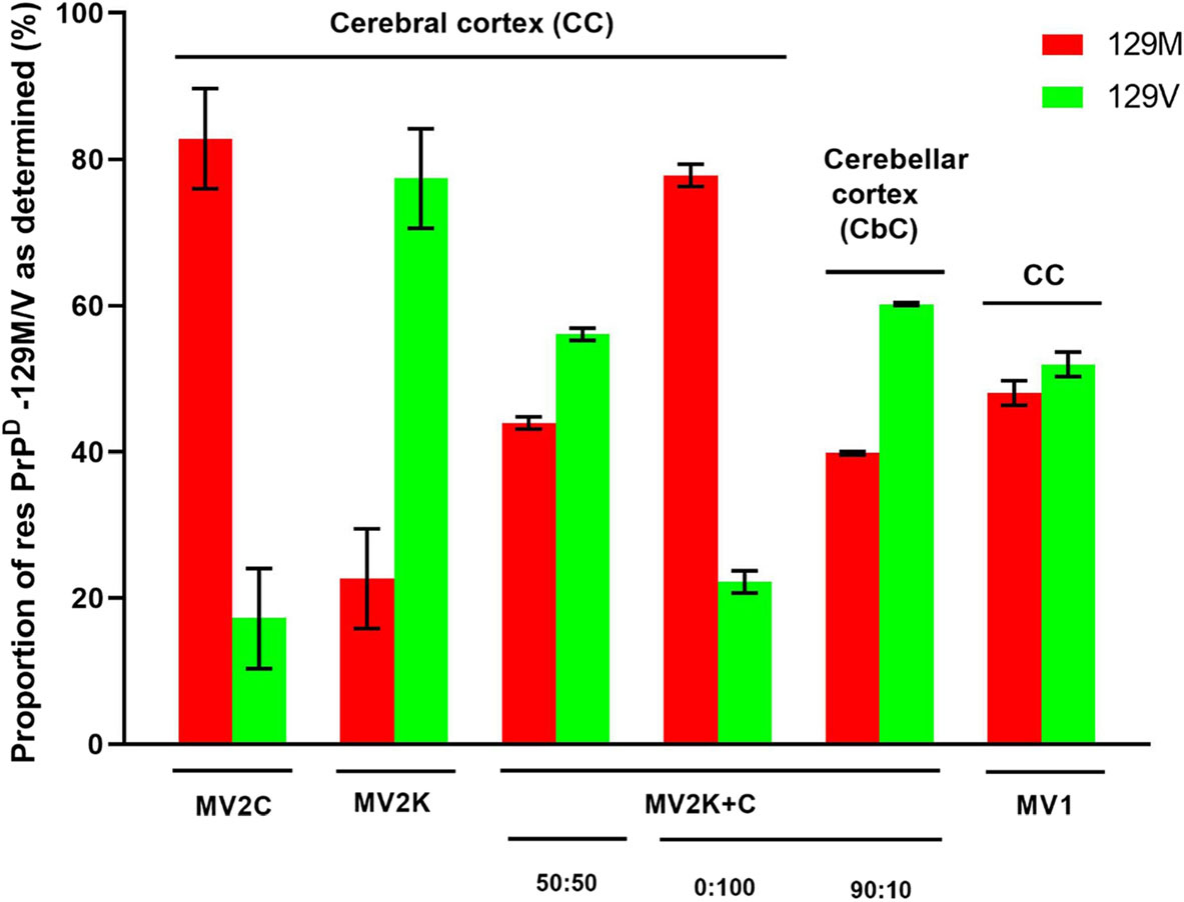
Relative abundance of 129 M and 129 V resPrP^D^ purified from distinct variants of sCJDMV. Relative proportions of both allotypic proteins in each case were determined by mass spectrometry using the spectral counting method. For the histotypically pure MV1, MV2C and MV2K variants data ± SEM are averages of three determinations for each of the nine total cases. For the mixed cases MV2K-C (~ 50:50) and MV2K-C 0:100 K:C in the cerebral and ~ 90:10 K:C in the cerebellar cortices, data are averages of three MS determination for each case. The fractions (%) of resPrP^D^-129 M in each MV2 variant and the MV1 subtype are: 83 ± 7% for MV2C, 23 ± 7% for MV2K, and 48 ± 2% for MV1. In pure MV2C and MV2K cases, the relative amounts of resPrP^D^-129 M and 129 V are significantly different (*p* < 0.0001 and 0.0002, respectively) whereas in MV1 the difference is not significant. In the two MV2K-C mixed cases, the resPrP^D^-129 M proportion is 44% for the CC ~ 50:50 first case; 78 and 40%, respectively, for the CC 0:100 and CbC ~ 90:10. As determined by one way ANOVA, *p* values for differences in relative populations of the two resPrP^D^ allotypic forms in distinct sCJD variants and subtypes are: MV2C vs. MV2K, *p* < 0.0001; MV1 vs. MV2C *p*  <0.009

We next determined the abundance resPrP^D^-129 M and -129 V in three pure cases of sCJD MV2C and MV2K (Fig. 4 and Additional File 5: Figure S3). Remarkably, MV2C, the histotype of which is virtually indistinguishable from that of MM2C, showed a large predominance of the 129 M allotype, with resPrP^D^-129 M accounting for 83 ± 7% of total resPrP^D^, a difference between populations of resPrP^D^-129 M and -129 V statistically highly significant (*p* < 0.0001). By contrast, a diametrically different ratio of the 129 M and 129 V allotypes was observed in sCJD MV2K cases, where resPrP^D^-129 M accounted for only 23 ± 7% of total resPrP^D^ (*p* < 0.0002 for the difference between populations of resPrP^D^-129 M and -129 V) (Fig. 4 and Additional File 5: Figure S3).

We also used the mass spectrometric method to assess the proportion of the resPrP^D^-129 M and -129 V in two distinct mixed MV2K-C sCJD cases. The first of these cases, in which the two histotypes were about evenly distributed throughout the cerebrum, was characterized by a comparable representation of resPrP^D^-129 M and -129 V (44 and 56%, respectively) (Fig. 4 and Additional File 5: Figure S3). The second variant harbored mixed, though anatomically segregated histotypes, with the MV2C histotype localized almost exclusively to the cerebrum and the MV2K histotype predominating in the cerebellum (Fig. 4 and Additional File 5: Figure S3). Consistent with distinct regional distribution of the two haplotypes, resPrP^D^-129 M and -129 V in the cerebral cortex of this mixed variant accounted for ~ 78 and 22% of total resPrP^D^, respectively, whereas the proportion of resPrP^D^-129 M and -129 V in the cerebellum was ~ 40 and 60%, respectively (Fig. 4 and Additional File 5: Figure S3).

A similar analysis was also undertaken for the sCJDMV1 subtype that shares the MM1 histotype (Fig. 1, panels s-x and Table 1). In this case, resPrP^D^-129 M and -129 V were represented in approximately equal proportions, accounting for ~ 48 ± 2 and 52 ± 2%, respectively of total resPrP^D^ (Fig. 4 and Additional File 5: Figure S3).

Finally, the identification of residue 129 and the N-terminus in the 20 and 19 kDa components of MV2K was also attempted. The single successful experiment aimed at determining the ratio of resPrP^D^-129 M and -129 V selectively in the 20 kDa fragment revealed the exclusive presence of methionine at position 129. Furthermore, three independent experiments identified G82, G86 and S97 N-termini, the first two of which are likely to be N-termini of the 20 kDa fragment, and the third of the 19 kDa component.

## Discussion

It is increasingly clear that successful treatment of prion diseases may need to be tailored to individual prion strain [13]. This notion underscores the need for the detailed understanding of the mechanisms of strain emergence and selection that lead to the heterogeneity of human prion diseases. This mechanistic insight will also likely have implications for development of therapeutic strategies in other neurodegenerative diseases that are characterized by prion-like propagation and structural polymorphism of misfolded protein aggregates.

Previous studies showed that both the PrP^D^ type 1 and 2 selection as well as phenotypic heterogeneity of sCJD are largely under the control of the 129 allotype (for review see [12, 34]). However, this scenario does not explain the phenotypic heterogeneity of the sCJDMV2 subtype, where the heterogeneity occurs without any variation of the 129 allotype and PrP^D^ type (Table 1).

To bridge this gap, here we have performed detailed characterization of different subtypes of sCJDMV cases, and for each of these cases we have used a mass-spectrometry based approach to determine the relative proportion of resPrP^D^-129 M and -129 V. This analysis revealed a novel, versatile mechanism by which residue 129 polymorphism of resPrP^D^ determines phenotypic heterogeneity, resulting in multiple variants within the MV2 subtype of sCJD. In particular, our data show that the occurrence of two variants, referred to as MV2C and MV2K, either with pure or mixed phenotypes, is directly related to the relative abundance of resPrP^D^-129 M and 129 V. The pure MV2C phenotype is characterized by large predominance of resPrP^D^-129 M. By contrast, in the pure MV2K phenotype, the predominant form of resPrP^D^ contains Val at position 129, with resPrP^D^-129 M accounting only for ~ 23% of total resPrP^D^. Furthermore, consistent with the notion that the nature of amino acid at position 129 (M or V) is a major determinant of disease phenotype in heterozygous cases of sCJD, we found that, in MV2K-C mixed phenotypes, the relative abundance of resPrP-129 M and -129 V correlates with the representation of the corresponding histotypes in these mixed cases. Of note, the second MV2K-C case shows that histotype-related quite different allotypic proportions of resPrP^D^ may occur in two separate regions of the same brain (in this case, cerebral and cerebellar cortices), suggesting that local mechanisms play a role in determining this topographic heterogeneity.

Different proportions of resPrP^D^ corresponding to two allotypes as determined in this study likely result from the preferential templated conversion of the normal form of PrP (PrP^C^) that matches the allotype of the dominant resPrP^D^. One cannot completely rule out the possibility that the observed different proportions of resPrP-129 M and -129 V in MV2C and MV2K cases result from differences in the resistances to PK digestion. However, this possibility is highly unlikely given that the MV2C and MV2K variants share PK titration profiles and conformational stabilities or both with the VV2 and MM2 subtypes, which, being 129 homozygous, are associated with PrP^D^ isoforms allotypically homogeneous.

Remarkably, even in the histotypically pure cases, neither resPrP^D^-129 M nor the resPrP^D^-129 V allotype accounted for 100% of total resPrP^D^, with the minor allotype always observed. Even though one cannot absolutely rule out the possibility that this apparent coexistence of both resPrP^D^ allotypes in pure phenotypes is due to a systematic error of our mass spectrometry-based measurements, this is rather unlikely. Thus, the presence of a small population (~ 20%) of resPrP^D^-129 V in MV2C cases appears to be an intrinsic property of this phenotype. This is very intriguing given the similarity of the MV2C and MM2 subtypes, both with regard to the histotype and resPrP^D^ conformational stability index. In this variant, PrP^C^-129 V might be converted into PrP^D^ that adopts the PrP^D^ MM2-like conformation and contributes to the MM2-like histotype. Alternatively, this minor resPrP^D^ component might remain “silent” with its histotype undetectable [4, 7].

The coexistence of resPrP^D^-129 V and -129 M (77:23 ratio) in the MV2K variant raises even more challenging questions, especially given the presence in this variant of two resPrP^D^ electrophoretic components of 19 kDa and 20 kDa. Data from our and other laboratories allowed for detailed characterization of these two components. With regard to the allotype, our sole successful attempt at sequencing the 20 kDa resPrP^D^ component indicated the presence of the M residue at position 129. However, transmission of the MV2K variant to knock-in 129 MM, MV and VV transgenic mice demonstrated that the 20 kDa component can be replicated only in the presence of at least one 129 M allele [16] . Altogether, these data identify the 20 kDa fragment as the sole component populating resPrP^D^-129 M in the MV2K subtype. Furthermore, further bioassays in 129 M allotypic mouse lines have shown that the 20 kDa resPrP^D^ fragment can also be replicated upon inoculation of VV2 sCJD prions (which lack the 20 kDa resPrP^D^ component) [16]. This observation suggests that the 20 kDa resPrP^D^ component in the MV2K subtype does not result from a direct templated conversion of 129 M PrP^C^ but is rather due to the adaptation of the VV2 19 kDa strain to the presence of the 129 M allele. Our sequencing efforts have identified residues G82 and G86 as N-termini of the 20 kDa resPrP^D^ component. This is consistent with the two most N-terminal residues that we observed in MV2 cases in a previous systematic sequencing study of different sCJD subtypes [29]. These two N-termini of the 20 kDa resPrP^D^ component differ from those previously identified (as residues G78 and G82) in typical type 1 resPrP^D^ associated with the MM1 phenotype [29]. Furthermore, our conformational stability data surprisingly indicate that, despite the immunoreactivity properties and N-terminus characteristics consistent with resPrP^D^ type 1, the 20 kDa resPrP^D^ species in the MV2K subtype appears to have conformational features of resPrP^D^ type 2, which significantly differ from those of all type 1 subtypes. Finally, a body of evidence indicates that the 20 kDa component of resPrP^D^ is the determinant of kuru plaque formation [6, 16] and [Cali et al. unpublished data]. Altogether, these features define PrP^D^ associated with the MV2K subtype as a unique prion strain in sCJD.

Based on the widely accepted notion that the MV1 variant is indistinguishable from the MM1 subtype, both with regard to the histotype as well as PrP^D^ and transmission characteristics, we expected the former variant to show higher proportion of resPrP^D^-129 M than resPrP^D^-129 V, as is the case for the MV2C variant. The finding of equal representation of the 129 M and 129 V allotypes in the MV1 variant raises questions as to the role of these two components in histotype determination. One plausible scenario would require PrP^C^-129 V to be converted into PrP^D^ with structural characteristics of PrP^D^-129 M type 1, participating in determination of the same histotype. Should this be the case, MV1 PrP^D^ would be the first example of human prion strain which, despite being comprised of similar amounts of two different resPrP^D^ allotypes, is associated with a homogeneous histotype.

Finally, it must be noted that our findings are at odds with the previous study of Moore and coworkers [20], who examined a similar case population using a similar mass spectrometry-based approach and found no systematic phenotype-specific differences between the populations of two resPrP^D^ isoforms. This is likely related to less stringent criteria used in the previous study for case selection, which could have resulted in the disproportionate representation of mixed (rather than pure) cases. Indeed, 10 out of the 14 sCJDMV cases examined in the previous study were mixed cases, either MV1-2C/K (4 out of 5 cases) or MV2K-C (6 out of 8 cases). Thus, it seems not surprising that the examination of a population of such mixed cases would lead to inconclusive results. However, when one considers only the “pure” cases used by Moore and colleagues, the relative proportions of the two resPrP^D^ isoforms appear to be comparable to those determined in the present work. Furthermore, all but one (possibly atypical or mixed) of the MV2K iatrogenic CJD cases classified in the study of Moore et al. as “pure” show the predominance of resPrP^D^ 129 V over 129 M. Therefore, applying more stringent case selection criteria, the findings by Moore et al. are consistent with ours.

## Conclusion

The mass spectrometry-based analysis of relative populations of resPrP^D^-129 M and -129 V allotype in extensively characterized cases of MV2C and MV2K variants, as well as cases of MV1, revealed a novel mechanism for phenotypic determination of human prion diseases. According to this mechanism, in MV2C, PrP^C^-129 M is preferentially converted to the disease-associated form, resulting in the predominance of resPrP^D^-129 M over resPrP^D^-129 V and leading to a relatively pure, MM2-like histotype. By contrast, in sCJDMV2K, there is a predominance of resPrP^D-^129 V, which has characteristics of type 2 and resembles resPrP^D^ associated with the VV2 subtype. ResPrP^D^-129 M in the MV2K variant is represented by a 20 kDa species, which likely results from the adaptation of the VV2 strain. Furthermore, also in the MV2K-C mixed variants, resPrP^D^ 129 allotypic proportions appear to correlate with the representations of the two histotypes. Finally, in the sCJDMV1 subtype that shares the histotype with the codon 129 homozygous MM1 subtype, resPrP^D^-129 M and -129 V were represented in approximately equal proportions.

## Supplementary information

**Additional file 1: Table S1.** Summary of conformational stability and PK resistance indices for individual subtypes and variants.

**Additional File 2: Table S2.** Peptides identified by mass spectrometry in tryptic digests of resPrP^D^ purified from sCJD MM2 and sCJD VV2 cases.

**Additional File 3: Figure S1.** Immunoblots of individual sCJD subtypes used to generate CSI and PK titration indexes. a and b: CSI and PK titration, respectively. Total PrP treated as indicated were quantified by infrared imaging of the electrophoretic band corresponding the unglycosylated isoforms; arrows: upper and lower arrows point to the unglycosylated 20 kDa and 19 kDa components of the MV2K- electrophoretic profile; single solid and dashed arrows identify the unglycosylated isoforms of resPrP^D^ types 2 and 1, respectively, of indicated sCJD variants and subtypes. Ab 3F4.

**Additional File 4: Figure S2.** Calibration of the mass spectrometric method for determination of relative concentrations of 129 M and 129 V PrP in mixtures containing different proportions of both proteins. a: Calibration data for recombinant human PrP (rhuPrP) using five different mixtures of 129 M and 129 V rhuPrP with concentrations of each protein determined by absorbance at 280 nm. b: Calibration data for resPrP^D^ purified from pure cases of sCJDMM2 and sCJDVV2 using five different proportions of 129 M and 129 V resPrP^D^ with concentrations of each protein determined by densitometric analysis of Western blots. In each case, the mixtures were digested with trypsin and analyzed by mass spectrometry. Spectral counting method was used to determine the relative proportions of 129 M and 129 V PrP polymorphs.

**Additional File 5: Figure S3.** Mass spectrometry data from the individual cases examined. The resPrP^D^ 129 M percentages for each of the 3 “pure” cases were: MV2C: 90.3, 88.9, 69.2; MV2K: 31.9, 26.7, 9.3; MV1: 45.2, 51, 47.9. The M:V proportions of the two MV2K-C mixed cases are reported in the legend of Fig. 4.

## Data Availability

The datasets used in the current study are available from the corresponding author on reasonable request.

## Abbreviations

Ab: Antibody
CSI: Conformational stability immunoassay
CC: Cerebral cortex
CbC: Cerebellar cortex
hr: Hour
MM: Homozygous methionine
min: Minutes
MS: Mass spectrometry
MV: Heterozygous methionine/valine
PK: Proteinase K
PrP^D^: Disease-related prion protein
sCJD: Sporadic Creutzfeldt-Jakob disease
sFI: Sporadic fatal insomnia
VV: Homozygous valine
