# Supplementary material for: A novel mechanism of phenotypic heterogeneity in Creutzfeldt-Jakob disease
Source: Acta Neuropathol Commun. 2020 Jun 19;8:85. doi: 10.1186/s40478-020-00966-x (PMC7304206; doi:10.1186/s40478-020-00966-x)
Supplement: Supplementary file 1 — Additional file 1: Table S1. Summary of conformational stability and PK resistance indices for individual subtypes and variants. [file 40478_2020_966_MOESM1_ESM.docx]

| **Additional File 1: Table S1**. Summary of conformational stability and PK resistance indices for individual subtypes and variants | | | |
| --- | --- | --- | --- |
| **sCJD Variant** | **Cases (N)** | **[GdnHCl]_1/2_ (M) ± SEM** | **[PK]_1/2_ (U/ml)**  **± SEM** |
| VV2 | 3/3^1^ | 1.3 ± 0.1 | 42 ± 5.1 |
| MM2 | 4 | 1.4 ± 0.06 | nd |
| MV2C |  | 1.6 ± 0.04 | nd |
| MV2K Tot | 3 | 1.1 ± 0.04 | nd |
| MV2K 19 kDa | 3/3^1^ | 1.1 ± 0.05 | 48 ± 2.3 |
| MV2K 20 kDa |  | 1.1 ± 0.03 | 49 ± 4.5 |
| MM1 | 4/3^1^ | 1.7 ± 0.03 | 17 ± 3.7 |
| MV1 | 3/3^1^ | 1.7 ± 0.06 | 13 ± 2.4 |
| ^1^The second number indicates the number of cases (N) for PK titration; nd: not determined. | | | |
