# Supplementary material for: A novel mechanism of phenotypic heterogeneity in Creutzfeldt-Jakob disease
Source: Acta Neuropathol Commun. 2020 Jun 19;8:85. doi: 10.1186/s40478-020-00966-x (PMC7304206; doi:10.1186/s40478-020-00966-x)
Supplement: Supplementary file 2 — Additional File 2: Table S2. Peptides identified by mass spectrometry in tryptic digests of resPrPD purified from sCJD MM2 and sCJD VV2 cases. [file 40478_2020_966_MOESM2_ESM.docx]

**Additional File 2: Table S2.** Peptides identified by mass spectrometry in tryptic digests of resPrP^D^ purified from sCJD MM2 and sCJD VV2 cases.

**
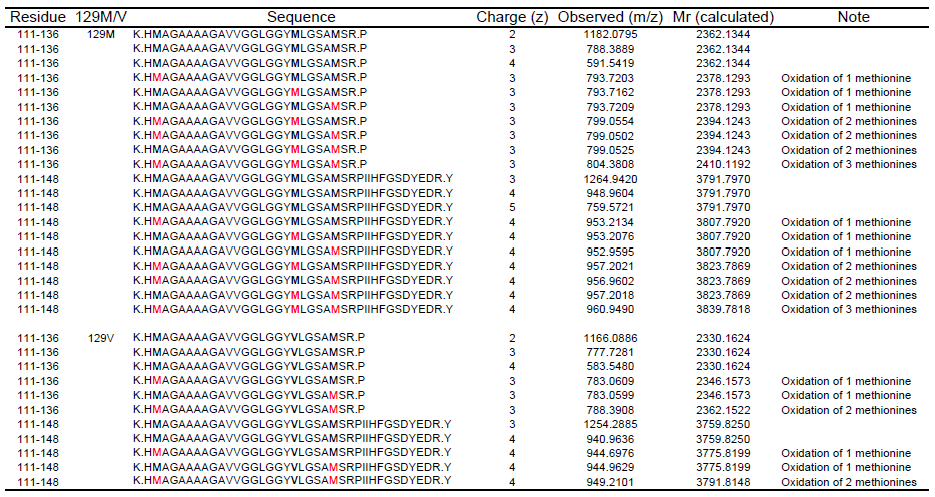
**

Residues 129M/V are shown in bold, oxidized methionine residues are identified in red.
