# Supplementary figures and images for: A novel mechanism of phenotypic heterogeneity in Creutzfeldt-Jakob disease
Source: Acta Neuropathol Commun. 2020 Jun 19;8:85. doi: 10.1186/s40478-020-00966-x (PMC7304206; doi:10.1186/s40478-020-00966-x)

**Figure S1**

**a**

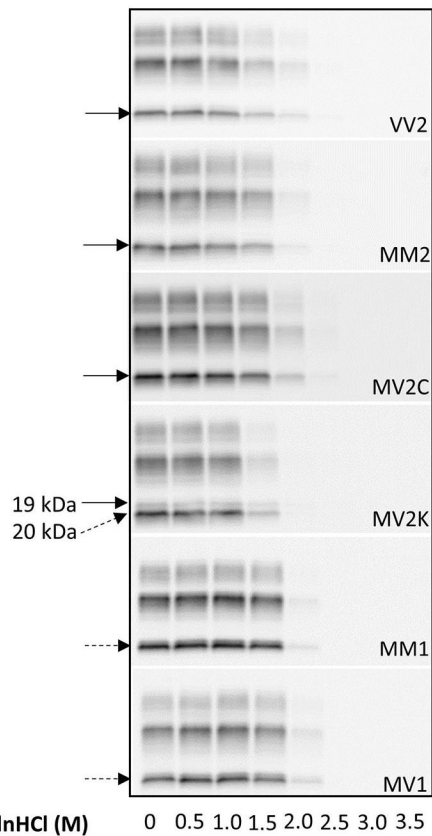

**b**

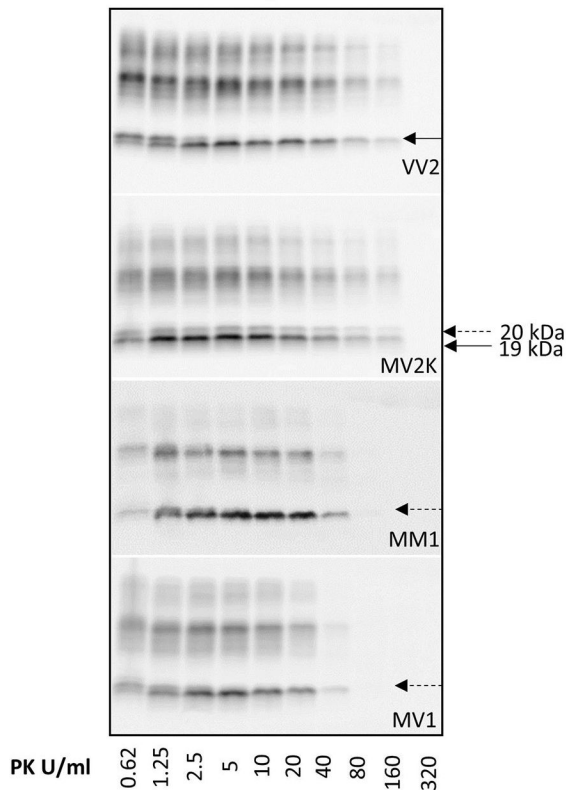

Supplement: Supplementary file 3 — Additional File 3: Figure S1. Immunoblots of individual sCJD subtypes used to generate CSI and PK titration indexes. a and b: CSI and PK titration, respectively. Total PrP treated as indicated were quantified by infrared imaging of the electrophoretic band corresponding the unglycosylated isoforms; arrows: upper and lower arrows point to the unglycosylated 20 kDa and 19 kDa components of the MV2K- electrophoretic profile; single solid and dashed arrows identify the unglycosylated isoforms of resPrPD types 2 and 1, respectively, of indicated sCJD variants and subtypes. Ab 3F4. [file 40478_2020_966_MOESM3_ESM.pdf]

**Figure S2**

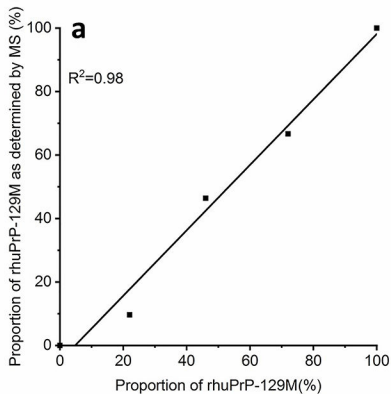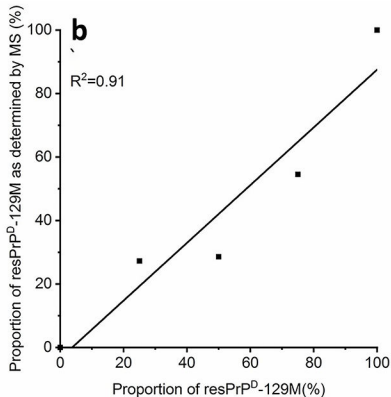

Supplement: Supplementary file 4 — Additional File 4: Figure S2. Calibration of the mass spectrometric method for determination of relative concentrations of 129 M and 129 V PrP in mixtures containing different proportions of both proteins. a: Calibration data for recombinant human PrP (rhuPrP) using five different mixtures of 129 M and 129 V rhuPrP with concentrations of each protein determined by absorbance at 280 nm. b: Calibration data for resPrPD purified from pure cases of sCJDMM2 and sCJDVV2 using five different proportions of 129 M and 129 V resPrPD with concentrations of each protein determined by densitometric analysis of Western blots. In each case, the mixtures were digested with trypsin and analyzed by mass spectrometry. Spectral counting method was used to determine the relative proportions of 129 M and 129 V PrP polymorphs. [file 40478_2020_966_MOESM4_ESM.pdf]

**Figure S3**

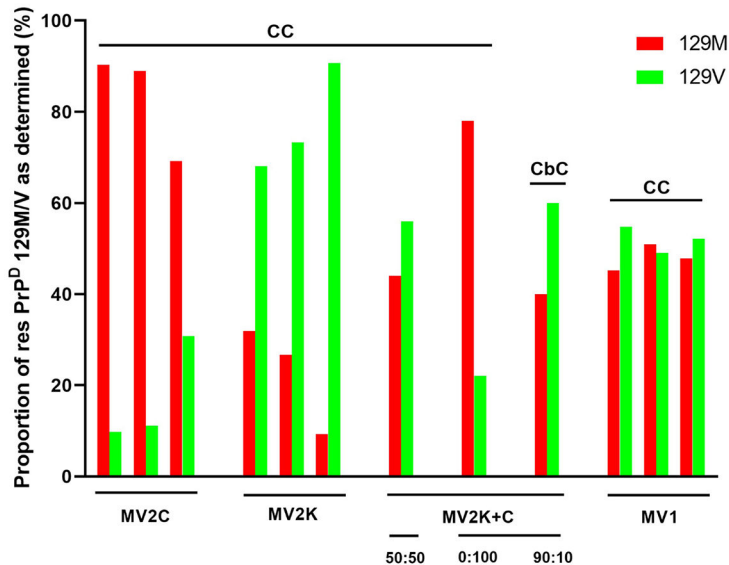

Supplement: Supplementary file 5 — Additional File 5: Figure S3. Mass spectrometry data from the individual cases examined. The resPrPD 129 M percentages for each of the 3 “pure” cases were: MV2C: 90.3, 88.9, 69.2; MV2K: 31.9, 26.7, 9.3; MV1: 45.2, 51, 47.9. The M:V proportions of the two MV2K-C mixed cases are reported in the legend of Fig. 4. [file 40478_2020_966_MOESM5_ESM.pdf]
